# Simultaneous learning and filtering without delusions: a Bayes-optimal combination of Predictive Inference and Adaptive Filtering

**DOI:** 10.3389/fncom.2015.00047

**Published:** 2015-04-30

**Authors:** Jan Kneissler, Jan Drugowitsch, Karl Friston, Martin V. Butz

**Affiliations:** ^1^Chair of Cognitive Modeling, Department of Computer Science, Faculty of Science, Eberhard Karls University of TübingenTübingen, Germany; ^2^Départment des Neurosciences Fondamentales, Université de GenèveGeneva, Switzerland; ^3^The Wellcome Trust Centre for Neuroimaging, Institute of Neurology, University College LondonLondon, UK

**Keywords:** predictive coding, Bayesian information processing, Kalman filtering, recursive least squares, illusions, forward model

## Abstract

Predictive coding appears to be one of the fundamental working principles of brain processing. Amongst other aspects, brains often predict the sensory consequences of their own actions. Predictive coding resembles Kalman filtering, where incoming sensory information is filtered to produce prediction errors for subsequent adaptation and learning. However, to generate prediction errors given motor commands, a suitable temporal forward model is required to generate predictions. While in engineering applications, it is usually assumed that this forward model is known, the brain has to learn it. When filtering sensory input and learning from the residual signal in parallel, a fundamental problem arises: the system can enter a delusional loop when filtering the sensory information using an overly trusted forward model. In this case, learning stalls before accurate convergence because uncertainty about the forward model is not properly accommodated. We present a Bayes-optimal solution to this generic and pernicious problem for the case of linear forward models, which we call Predictive Inference and Adaptive Filtering (PIAF). PIAF filters incoming sensory information and learns the forward model simultaneously. We show that PIAF is formally related to Kalman filtering and to the Recursive Least Squares linear approximation method, but combines these procedures in a Bayes optimal fashion. Numerical evaluations confirm that the delusional loop is precluded and that the learning of the forward model is more than 10-times faster when compared to a naive combination of Kalman filtering and Recursive Least Squares.

## 1. Introduction

There is wide agreement that a major function of the brain is to generate predictions about future events based on observations made in the past. This predictive coding principle is now considered by many as the universal guiding principle in explaining the majority of brain activities (Rao and Ballard, 1999; Friston, 2003; König and Krüger, 2006; Kilner et al., 2007; Bar, 2009). Friston et al. (2006) expands this framework under a free-energy principle, which can also explain action selection by considering the (desired) effects of actions on the sensory inputs (cf. also Friston, 2010). Indeed, the free-energy principle entails the Kalman filter and many other learning, adaptation, and inference schemes under appropriate forward or generative models (Friston, 2009, 2010). In this paper, we derive a Bayes-optimal scheme for learning a predictive, forward velocity (kinematics) model and simultaneously using this model to filter sensory information. The resulting scheme effectively combines predictive encoding with the learning of a forward model by carefully separating system state estimates from the encoding of the forward model.

A large portion of the variability that we encounter in our sensory inputs can be directly attributed to our motor activities (movements of the parts of the body, self propulsion, saccades, uttered sounds, etc.). The existence of neural pathways that send *efference copies* of motor commands back to sensory areas and other regions has been confirmed in primates but also in many species with much simpler nervous systems. Helmholtz (1925) coined the term *corollary discharge* for this feedback loop relaying motor outputs from motor areas to other brain regions (cf. also Sperry, 1950 and a recent review by Crapse and Sommer, 2008). Corollary discharge represents the physiological basis for the reafference principle of von Holst and Mittelstaedt (1950), stating that self-induced effects on sensory inputs are suppressed and do not lead to the same level of surprise or arousal as exafferent stimulation. This has been interpreted by Blakemore et al. (2000) for the curious fact that we are not able to tickle ourselves. Failures of the efference copy mechanism have been proposed as a basis for some schizophrenic symptoms (Pynn and DeSouza, 2013). It has been argued whether the stability of the visual percept—despite the perpetual movements of the eye balls—relies on efference copies (Sommer and Wurtz, 2006), or if other mechanism play the crucial role (Bridgeman, 2007).

The suppression of sensory information related to one's own motor actions has great similarity to the way in which noise is suppressed in Kalman filtering—a technique developed by Kalman (1960), which has an enormous range of technical applications. The basic approach of Kalman filtering is to interpolate between the new measurement and predictions of the new state based on its estimate in the previous time step. The mixing coefficient (Kalman gain) is adapted online and represents a balance between confidence in the prediction and the precision (or reliability) of new information. Assumptions about the nature of sensory noise allow to optimally determine the mixing coefficient using Bayesian information fusion. It has been demonstrated in several contexts that the brain can perform Bayes-optimal fusion of information sources with different precision (Ernst and Banks, 2002; Körding and Wolpert, 2004). It can be assumed that (amongst other uses) the information of corollary discharges is employed to optimize the information gain supplied by sensory feedback (in terms of a Kalman gain).

However, unlike in engineered systems, in biological systems the relationship between motor commands and their sensory consequences is not known a priori. The brain has to learn and continuously adapt this mapping. This mapping from motor commands to state changes is called *forward velocity kinematics*. In general, forward velocity kinematics can take the form of a highly non-linear but nevertheless smooth function, which may be approximated adequately by locally linear maps. Learning the kinematics thus amounts to a regression task within each local approximator.

It can be proven (under mild assumptions) that the optimal linear unbiased regression estimator is given by the least squares approach that dates back to Gauss (1821). An online, iterative version called recursive least squares (RLS) was developed by Plackett (1950). It might thus appear that a straightforward combination of RLS with Kalman filtering could easily solve the problem of learning the forward model, while filtering sensory input. Our previous work (Kneissler et al., 2012, 2014) has shown that the combination can indeed accelerate learning when compared with RLS-learning given unfiltered sensory information. To perform optimal Bayesian information fusion, the precision of the predicted state (relative to sensory information) has to be estimated. This estimate, however, is influenced by the filtering implicit in the previous time steps. If the sensory signal was too strongly filtered by the prediction, an overly strong confidence in the predictions can develop. As a result, the system falls into a delusional state due to unduly high self-confidence: ultimately, in this case, the system will completely ignore all new incoming information.

The contribution of this paper is to provide a rigorous (Bayes optimal) mathematical basis for learning a linear motor-sensor relationship and simultaneously using the learned model for filtering noisy sensory information. Formally, our method becomes equivalent to a joint Kalman filter (Goodwin and Sin, 1984), in which both states and the forward model are learned and tracked simultaneously by a global Kalman filter; thereby solving a dual estimation problem. In contrast to previous applications of this approach, however, we derive separate, interacting update equations for both state estimation and the forward model, thus making their interaction explicit. We empirically confirm that the ensuing Predictive Inference and Adaptive Filtering (PIAF) does not fall into self-delusion and speeds-up learning of the forward model more than 10-fold, when compared to naive RLS learning combined with Kalman filtering.

In Section 2, we provide a mathematical formulation of the problem, outline the derivation of the solution (details are given in the Supplementary Material) and present the resulting update equations. In Section 3 we mathematically detail the relation of PIAF to a joint generalization of Kalman filtering and RLS. Finally, in Section 4 we present experimental results comparing PIAF with several other possible model combinations, confirming robust, fast, and accurate learning. A discussion of implications and future work conclude the paper.

## 2. Methods

Our formulation assumes the presence of a body or system with particular, unobservable system states *z_n_* at a certain iteration point in time *n* (see Table 1 for an overview of mathematical symbols). The body state can be inferred by sensors, which provide noisy sensory measurements *x_n_*. Formally, each of the measurements relates to the system state by

(1) $$x_{n}=z_{n}+\epsilon_{s,n},$$

where ϵ_*s,n*_ ~ 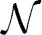 (0, σ^2^_*s*_) is Gaussian sensory noise with zero mean and sensory noise variance of inverse precision, σ^2^_*s*_. Clearly, the smaller this variance is, the more precise information each measurement reveals about the true system state.

**Table 1:** **Mathematical symbols used**.

*z_n_*	System state at time step *n* (scalar)
*x_n_*	Sensor measurement at time step *n* (scalar)
$\dot{q}$_*n*_	Motor command at time step *n* (vector)
***w***	Linear model mapping motor commands to state change (vector)
σ_*s*_	Standard deviation of sensor noise (scalar)
σ_*p*_	Standard deviation of process noise (scalar)
μ_*z*_	Mean of state estimate (scalar)
μ_*w*_	Mean of control parameter estimate (vector)
Σ_*zz*_	Variance of state estimate (scalar)
Σ_*ww*_	Covariance of control parameter estimate (matrix)
Σ_*zw*_	Covariance between state and control parameter estimate (vector)

We further model body or system control by iterative motor commands. Formally, we assume that at each point *n* in time a motor command $\dot{q}$_*n*_ is executed, causing a noisy change of the system state that is linearly related to the motor command by

(2) $$z_{n}=z_{n-1}+\;{\dot{q}}_{n}^{T}w+\epsilon_{p,n}.$$

In the above, the motor command $\dot{q}$_*n*_ is a *D_q_*-dimensional vector, and its effect on the system state is modulated by the unknown control parameter ***w*** of the same size. Additionally, zero-mean Gaussian process noise ϵ_*p,n*_ ~ 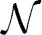 (0, σ^2^_*p*_) with variance σ^2^_*p*_ perturbs the state transition. This noise captures imperfections in the motor command execution, as well as possible deviations from linearity.

**Figure 1:**
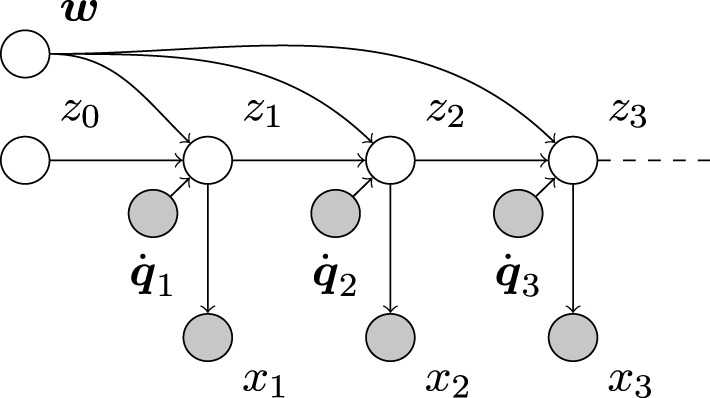
**Directed acyclic graph (e.g., Bishop, 2006) of our system model**. Empty and filled circles represent unobserved and observed random variables, respectively, and arrows indicate dependencies between them. As can be seen, the unobserved system state sequence *z*_0_, *z*_1_, *z*_2_, … depends on both the observed sequence of motor commands $\dot{q}$_1_, $\dot{q}$_2_, …, and the unobserved control parameters ***w***. The sensory measurements, *x*_1_, *x*_2_, …, only depend on the corresponding system state.

Overall, this results in the complete system model (Figure 1)

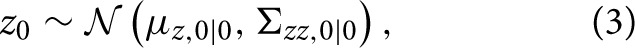

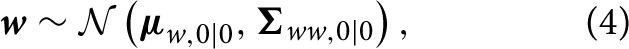

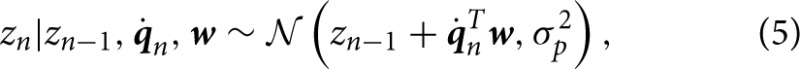

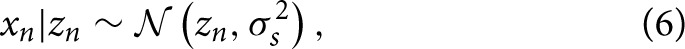









where μ_*z*,0|0_, Σ_*zz*,0|0_, μ_*w*,0|0_, and Σ_*ww*,0|0_ are prior parameters and the control command $\dot{q}$_*n*_ as well as the sensory signal *x_n_* are the observables at each iteration. In terms of notation, ·_.,*n*|*n*−1_ denotes the prediction prior after having measured *x*_1:*n*−1_ and having applied motor commands $\dot{q}$_1:*n*_, but before measuring *x_n_*. Once this *x_n_* is measured, the posterior parameters change to ·_.,*n*|*n*_.

Technically, the problem of estimating or learning the parameters of a forward model, while estimating states is a dual estimation problem—estimating both, the system states *z_n_* as well as the forward model parameters ***w***. Crucially, the (Bayes) optimal state estimates need to accommodate uncertainty about the parameters and vice versa. This means one has to treat the dual estimation problem using a single model inversion—such that conditional dependencies between estimates of states and parameters are properly accommodated. Heuristically, this means that the precision (inverse variance) assigned to state prediction errors has to incorporate uncertainty about the parameters (and vice versa). In what follows, we will illustrate how the optimum precision (implicit in the Kalman gain that is applied to prediction errors) can be augmented to accommodate this uncertainty. We show that a rigorous mathematical treatment of the dual estimation problem using Bayesian inference leads to a stable learning and filtering algorithm, using the forward model knowledge to filter sensory information and concurrently using the resulting residual for optimizing the forward model online.

The optimal way to identify the control parameters ***w*** and, simultaneously, the sequence of system states, *z*_1_, *z*_2_, …, corresponds to updating the joint posterior belief *p* (*z_n_*, ***w***|*x*_1:*n*_, $\dot{q}$_1:*n*_) over both quantities with every applied motor command $\dot{q}$_*n*_ and measurement *x_n_*, where we have used the shorthand notation *x*_1:*n*_ = {*x*_1_, … *x_n_*} and $\dot{q}$_1:*n*_ = {$\dot{q}$_1_, …, $\dot{q}$_*n*_}. According to the assumption of the Markov property and due to the linear Gaussian structure of our system model, this posterior will be jointly Gaussian, such that we can parameterize it by

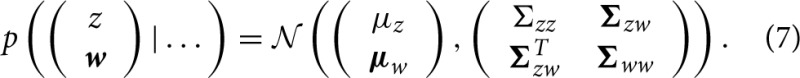



In the above, μ_*z*_ and Σ_*zz*_ is the mean and variance of our belief about the system state *z*, and μ_*w*_ and Σ_*ww*_ the mean vector and covariance of our belief about the control parameters ***w***. Furthermore, Σ_*zw*_ is a *D_q_*-element row vector that denotes the covariance between *z* and ***w***. It essentially captures the belief about how these two quantities are related.

Note that generally one could apply a global Kalman filter to jointly track *z* and ***w***, leading to what is known as a *joint Kalman filter* over an augmented state space (Goodwin and Sin, 1984). However, this would obfuscate the interaction between simultaneously tracking the system's state and inferring its forward model parameters. For this reason, we derive the update equations for the posterior over *z* and ***w*** separately, thus making their interactions explicit (and enforcing our a-priori beliefs that the true forward model parameters do not change with time).

In the following, we describe how the posterior parameter estimates change once a motor command $\dot{q}$_*n*_ is applied and we measure *x_n_*. As in the standard Kalman filter, this change is decomposed into a *prediction step*, which relates to the prediction of *z_n_* based on our belief about *z*_*n*−1_ and the applied motor command $\dot{q}$_*n*_, and an *update step*, which updates this prediction in the light of the measured *x_n_*. Moreover, an additional *update step* is necessary to infer the new forward model parameter ***w*** estimates.

### 2.1. Adaptive filtering: prediction step

Before the prediction step, we assume to have measured *x*_1:*n*−1_ after applying motor commands $\dot{q}$_1:*n*−1_. At this point, we have formed a posterior belief *p* (*z*_*n*−1_, ***w*** |μ_*n*−1|*n*−1_, Σ_*n*−1|*n*−1_) that decomposes into *z*_*n*−1_ and ***w***-related components as in Equation (7). The prediction step describes how the posterior parameters change in the light of applying a motor command $\dot{q}$_*n*_, which leads to a transition in the system state from *z*_*n*−1_ to *z_n_*, but before measuring the new system state via *x_n_*.

Computing the requisite first and second-order moments of *z* after the transition step (see Supplementary Material) results in the following computation of the updated prior belief about *z_n_*:

(8) $$\mu_{z,n|n-1}=\;\mu_{z,n-1|n-1}+\;{\dot{q}}_{n}^{T}\mu_{w,n-1|n-1},$$

(9) $$\begin{array}{l} \Sigma_{zz,n|n-1}=\Sigma_{zz,n-1|n-1}+\sigma_{p}^{2}+{\dot{q}}_{n}^{T}\Sigma_{ww,n-1|n-1}{\dot{q}}_{n}\\ \;+2\Sigma_{zw,n-1|n-1}{\dot{q}}_{n}. \end{array}$$

As can be seen, the mean parameter is updated in line with the system state transition model, Equation (2). The variance (inverse precision) parameter accommodates the process noise ϵ_*p,n*_ through σ^2^_*p*_ and, through the remaining terms, our uncertainty about the control parameters ***w*** and how it relates to the uncertainty about the system state *z_n_*. Due to the uncertainty in the control model and the process noise, the prior prediction of *z_n_* will always be less precise than the posterior belief about *z*_*n*−1_.

Moreover, a change in the state *z_n_* changes how ***w*** and *z_n_* are correlated, which is taken into account by

(10) $$\Sigma_{zw,n|n-1}=\Sigma_{zw,n-1|n-1}+\;{\dot{q}}^{T}\Sigma_{ww,n-1|n-1}.$$

This completes the adaptive filtering parameter updates for the prediction step.

### 2.2. Adaptive filtering: update step

In the update step, we gain information about the prior predicted system state *z_n_* by measuring *x_n_*. By Bayes' rule (see Supplementary Material), this leads the parameters describing the belief about *z_n_* to be updated by

(11) $$\mu_{z,n|n}\;=\mu_{z,n|n-1}+\frac{\Sigma_{zz,n|n-1}}{\sigma_{s}^{2}+\Sigma_{zz,n|n-1}}(x_{n}-\mu_{z,n|n-1}),$$

(12) $$\Sigma_{zz,n|n}\;=\frac{\sigma_{s}^{2}\Sigma_{zz,n|n-1}}{\sigma_{s}^{2}+\Sigma_{zz,n|n-1}}.$$

In the above, the mean parameter is corrected by the prediction error *x_n_* − μ_*z,n*|*n*−1_ in proportion to how our previous uncertainty Σ_*zz,n*|*n*−1_ relates to the predictive uncertainty σ^2^_*s*_ + Σ_*zz,n*|*n*−1_ about *x_n_*. Thus, the belief update accounts for deviations from our predictions that could arise from a combination of our uncertainty about the control parameters ***w*** and the process noise ϵ_*p,n*_. This update is guaranteed to increase our certainty about *z_n_*, which is reflected in a Σ_*zz,n*|*n*_ that is guaranteed to be smaller than Σ_*zz,n*|*n*−1_ before having observed *x_n_*. Note that the ratio of variances in Equations (11, 12) corresponds to the Kalman gain and represents a Bayes optimal estimate of how much weight should be afforded the (state) prediction errors.

In parallel, the covariance of our belief about ***w*** is updated and the mapping is re-scaled by:

(13) $$\Sigma_{zw,n|n}=\frac{\sigma_{s}^{2}}{\sigma_{s}^{2}+\Sigma_{zz,n|n-1}}\Sigma_{zw,n|n-1},$$

to reflect the additional information provided by *x_n_*. Thus, the a-posteriori state expectations μ_*z,n*|*n*_ and covariances Σ_*zz,n*|*n*_ and Σ_*zw,n*|*n*_ are determined.

### 2.3. Predictive inference: prediction and update step

Predictive inference adjusts the forward model control parameters ***w***.

Applying a motor command reveals nothing about the control parameters ***w*** and its parameters remain unchanged,

(14) $$\mu_{w,n|n-1}=\mu_{w,n-1|n-1},\;\Sigma_{ww,n|n-1}=\Sigma_{ww,n-1|n-1}.$$

The control parameter priors are thus equal to the previous posteriors.

The measured state information *x_n_*, on the other hand, provides information about the control parameters ***w***, leading to the following parameter updates:

(15) $$\mu_{w,n|n}\;=\mu_{w,n|n-1}+\frac{\Sigma_{zw,n|n-1}^{T}}{\sigma_{s}^{2}+\Sigma_{zz,n|n-1}}(x_{n}-\mu_{z,n|n-1}),$$

(16) $$\Sigma_{ww,n|n}\;=\Sigma_{ww,n|n-1}-\frac{1}{\sigma_{s}^{2}+\Sigma_{zz,n|n-1}}\Sigma_{zw,n|n-1}^{T}\Sigma_{zw,n|n-1}.$$

Its expectation is, as the mean estimate associated with *z_n_*, modulated by the prediction error *x_n_* − μ_*z,n*|*n*−1_. This prediction error is mapped into a prediction error about ***w*** by multiplying it by (an appropriately re-scaled) Σ_*zw,n*|*n*−1_, which is our current, prior best guess for how *z_n_* and ***w*** depend on each other. Thus, mean and variance estimates of the control parameters are updated, taking into account the residual between the measurement and the prior state estimate (*x_n_* − μ_*z,n*|*n*−1_), the certainty in the measurement σ^2^_*s*_, the prior certainty in the state estimate Σ_*zz,n*|*n*−1_, and the prior covariance estimate between state estimate and control parameters Σ_*zw,n*|*n*−1_.

If one examines (Equations 11, 12, 15, 16) one can see a formal similarity, which suggests that both states and parameters are being updated in a formally equivalent fashion. In fact, one approach to parameter estimation in the context of Kalman filtering is to treat the parameters as auxiliary states that have no dynamics. In other words, one treats the parameters as hidden states that have constant (time invariant) values. Goodwin and Sin (1984) exploit this in their *joint Kalman filter* technique. However, as intimated above, this approach obscures the different nature of the system's state and forward model parameters, while our approach clearly separates the two. Technically, this separation can be interpreted as an extended form of mean field approximation that matches both mean and variance of the posterior probability distribution over the states and forward model parameters. As the exact posterior is Gaussian, matching these two moments causes this approximation to perfectly coincide with the exact solution. However, the interpretation of this solution might guide approaches to handle non-linear rather than linear relations between control and state changes. This may be especially important if we consider the scheme in this paper as a metaphor for neuronal processing.

### 2.4. Illustration of information flow

**Figure 2:**
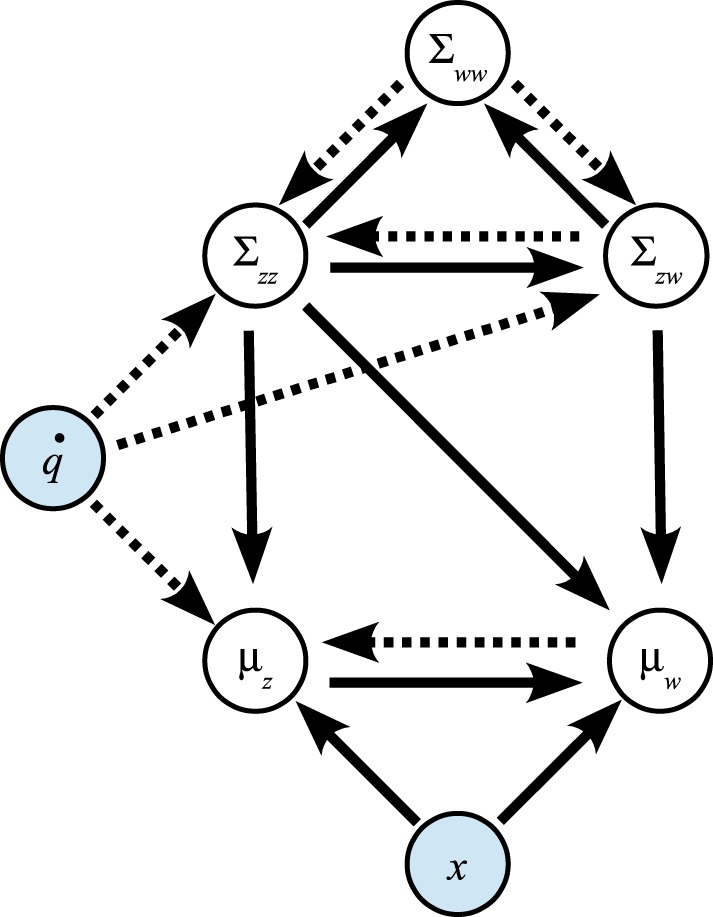
**Influence of variables on each other in prediction step (dotted arrows) and update step (solid arrows)**.

To clarify the interaction between adaptive filtering and predictive inference further, the Bayesian graph in Figure 2 shows the paths along which the information estimates and certainties influence each other.

In the prediction step of the adaptive filtering component, information is transferred from μ_*w*_ to μ_*z*_ and along Σ_*ww*_ → Σ_*zw*_ → Σ_*zz*_. In the update step of the adaptive filtering component, this information flow is reversed: the issued control signal $\dot{q}$ leads to updates of the variance estimate Σ_*zz*_, the covariance Σ_*zw*_, and the state estimate μ_*z*_ in the adaptive filtering component.

Moreover, in the predictive inference component, the residual (*x_n_* − μ_*z,n*|*n*−1_) leads to updates of the control parameter means μ_*w*_ as well as the covariance estimate Σ_*ww*_.

## 3. Relation to Kalman filter and RLS

The method described above describes how to track the state while simultaneously inferring the control parameters in a Bayes optimal sense. In this section, we show how our approach relates to uncoupled (naive) Kalman filtering for state tracking and RLS for parameter inference.

### 3.1. Relation to Kalman filter

**Figure 3:**
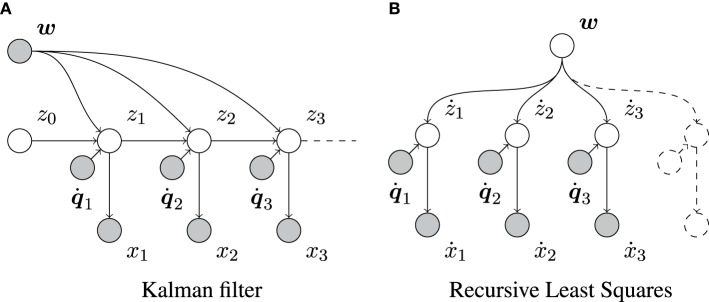
**Graphical representation of the Kalman filter and the RLS algorithm**. Empty and filled circles represent unobserved and observed random variables, respectively, and arrows indicate dependencies between them. **(A)** Shows that, in contrast to our full model (Figure 1), the Kalman filter assumes the control parameters ***w*** to be known. **(B)** Shows that, in contrast to our model (Figure 1), RLS does not take the sequential dependencies between consecutive changes in *x* into account. Here, ẋ_*n*_ = *x_n_* − *x*_*n*−1_ and ż_*n*_ = *z_n_* − *z*_*n*−1_.

We can relate the above to the Kalman filter by assuming that the control parameters ***w*** are known rather than inferred (Figure 3A). Then, the only parameters to be updated are μ_*z*_ and Σ_*zz*_.

In this case, the prediction step is given by (see Supplementary Material)

(17) $$\mu_{z,n|n-1}=\mu_{z,n-1,n-1}+\;{\dot{q}}_{n}^{T}w,$$

(18) $$\Sigma_{zz,n|n-1}=\Sigma_{zz,n-1|n-1}+\sigma_{p}^{2}.$$

The only different to the prediction step of our model, Equations (8, 9), is that ***w*** now replaces Equation (17) μ_*w*_ and Σ_*zz*_ does not include the uncertainty about ***w*** in its update.

The update step for *z_n_* is found by Bayes' rule (see Supplementary Material), resulting in

(19) $$\mu_{z,n|n}\;=\mu_{z,n|n-1}+\frac{\Sigma_{zz,n|n-1}}{\sigma_{s}^{2}+\Sigma_{zz,n|n-1}}(x_{n}-\mu_{z,n|n-1}),$$

(20) $$\Sigma_{zz,n|n}\;=\frac{\sigma_{s}^{2}\Sigma_{zz,n|n-1}}{\sigma_{s}^{2}+\Sigma_{zz,n|n-1}}$$

Thus, the update step for μ_*z*_ and Σ_*zz*_ remains unchanged (cf. Equations 11, 12), showing that the main difference to the full model is the lack of considering the uncertainty in the estimate of ***w***.

### 3.2. Relation to recursive least squares

As was shown elsewhere, RLS is a special case of the Kalman filter with a stationary state (e.g., Murphy, 2012). It does not consider sequential dependencies between successive system states (compare Figures 1, 3B). RLS is very suitable for estimating ***w*** by transforming the transition model, *z_n_* = z_*n*−1_ + $\dot{q}$*^T^_n_**w*** + ϵ_*p*_ (Equation 2) into ż_*n*_ = *z_n_* − *z*_*n*−1_ = $\dot{q}$*^T^_n_**w*** + ϵ_p_, in which the different ż_1_, ż_2_, … are, in fact, independent. In its usual form, RLS would assume that these ż_*n*_'s are observed directly, in which case its estimate of ***w*** would be Bayes-optimal. However, the ż_*n*_'s are in our case only observable through ẋ_*n*_ = *x_n_* − *x*_*n*−1_ = ż_*n*_ + ϵ_*s,n*_ − ϵ_*s,n*−1_. Furthermore, two consecutive ẋ_*n*_ and ẋ_*n*+1_ are correlated as they share the same sensory noise ϵ_*s,n*_. Therefore, RLS applied to our problem suffers from two shortcomings. First, the sensory noise appears twice in each RLS “observation” ẋ_*n*_. Second, the observations are correlated, contrary to the assumptions underlying RLS.

In terms of update equations, RLS applied to ẋ_1_, ẋ_2_, … features the same prediction step as the full model (see Supplementary Material), except for the covariance terms Σ_*zz*_ and Σ_*zw*_, which are updated according to

(21) $$\Sigma_{zz,n|n-1}=\Sigma_{zz,n-1|n-1}+\sigma_{p}^{2}+\;{\dot{q}}_{n}^{T}\Sigma_{ww,n-1|n-1}{\dot{q}}_{n},$$

(22) $$\Sigma_{zw,n|n-1}=\;{\dot{q}}_{n}^{T}\Sigma_{ww,n-1|n-1}.$$

Compared to Equations (9, 10), this prediction step effectively assumes Σ_*zw,n*−1|*n*−1_ = 0, which reflects RLS's independence assumption about consecutive observations. The RLS update step is the same as for the full model, see Equations (11–13, 15, 16); however, σ^2^_*s*_ are now replaced by the inflated variance 2σ^2^_*s*_.

## 4. Results

The complete scheme for Predictive Inference and Adaptive Filtering (PIAF), which is given by Equations (8–13), was tested numerically using one-dimensional control signals $\dot{q}$_*n*_. We assumed a constant, but unknown proportionality factor *w* = 1 (the prior estimate was set to μ_*w*,0|0_ = 0).

In one series of experiments, we applied a continuous control of the form $\dot{q}$_*n*_ = ω · cos(ω · *n* + ϕ)with $\omega =\frac{2\pi }{T}$, where the period was chosen *T* = 50 time steps. The starting state was set to *z*_0_ = sin(ϕ) such that the resulting signal *z_n_* oscillates periodically between −1 and 1. The start phase ϕ was chosen randomly, without giving PIAF any information about it (μ_*z*,0|0_ = 0). The variance priors were chosen as follows: Σ_*zz*,0|0_ = 10^4^ (range of unknown signal), Σ_*ww*,0|0_ = 1 (control commands and signal have same order of magnitude), Σ_*zw*,0|0_ = 0 (prior covariance is a diagonal matrix).

In the second set of experiments the control commands $\dot{q}$_*n*_ where sampled randomly from the Gaussian distribution 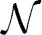 $\left(0,\frac{1}{2}\omega^{2}\right)$ with same mean and variance like the sinusoidal control in the first set of experiments.

In all experiments presented in this paper, the standard deviation of the sensory noise was set to σ^2^_*s*_ = 4 corresponding to an MSE signal-to-noise ratio of 1 : 8 (with respect to *z*).

**Figure 4:**
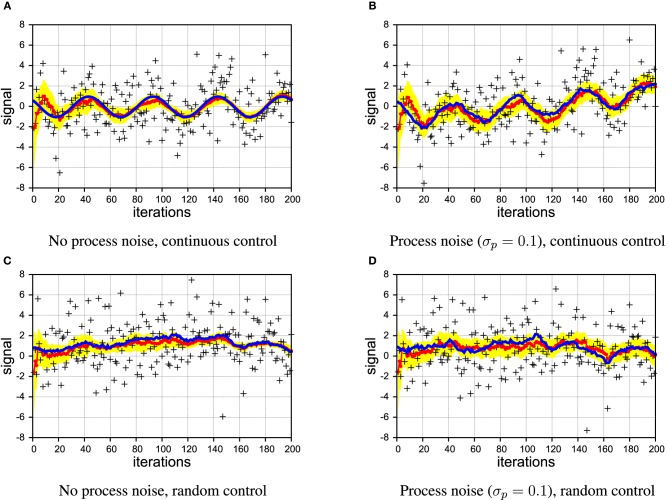
**Example of filtering showing the true signal (blue line), samples (black crosses), estimated signal (red line with dots), and the 2σ confidence interval (area shaded in yellow)**. [σ_*s*_ = 2 in all panels]. **(A,B)** continuous control; **(C,D)** random control; **(A,C)** without process noise; **(B,D)** with process noise.

As Figure 4A shows, PIAF converged very quickly to a good estimate close to the true signal in the absence of process noise. Despite the large amount of noise present in the samples, the deviation of the estimated signal is below 0.1 after a few 100 time steps. In Figure 4B, the experiment was repeated with a considerable level of process noise (σ_*p*_ = 0.1). The estimated signal is struggling to keep up with the jittery signal, which is reflected in the estimated variance, which does not decrease further after the first couple of steps. Nonetheless, the variance estimates appear warranted as the shaded area of width ±2 σ_*z*_ around the estimate μ_*z*_ encloses the true signal.

In Figures 4C,D, we illustrate that also in the case of irregular, random control, PIAF is working fine when there is no process noise. In the case with process noise (σ_*p*_ = 0.1), it produces an “apparently reasonable” posterior variance estimate (we investigate this quantitatively further in Section 4.3).

### 4.1. Dependence of performance on process noise

**Figure 5:**
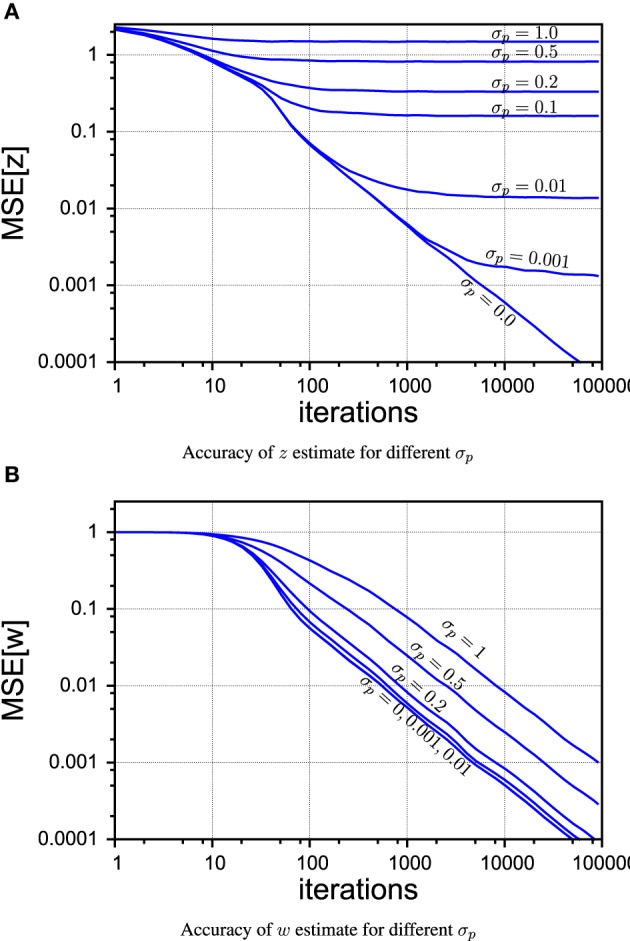
**PIAF accuracy for different settings of σ_*p*_ in the case of continuous control. (A)** Mean squared error of μ_*w*_ **(B)** mean squared error of μ_*z*_ [σ_*s*_ = 2, σ_*p*_ = 0, 0.001, 0.01, 0.1, 0.2, 0.5, 1].

In Figure 5 we compare different levels of process noise. There is no qualitative difference between continuous and random control commands in this experiment, so we show only the plots for the case of the sinusoidal control signals. The *z*-estimate, shown in Figure 5A, is obviously limited by the amount of process noise that contaminates the true signal at every time step. Nevertheless, as it can be seen in Figure 5B, PIAF can estimate *w* well for moderate process noise levels.

### 4.2. Comparing with RLS

**Figure 6:**
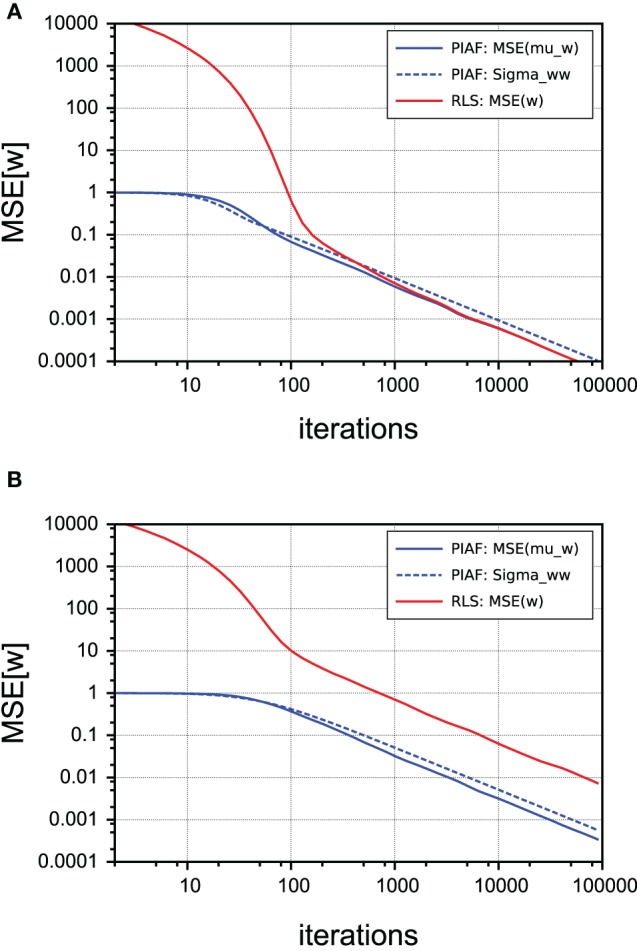
**Comparison of performance of *w*-estimation provided by PIAF (blue) and RLS (red)**. The accuracy is measured using the mean squared error. The variance estimate σ^2^_*w*_ of PIAF is also shown (dashed line). [σ_*s*_ = 2, σ_*p*_ = 0.1]. **(A)** Continuous control; **(B)** Random control.

In order to compare the performance of PIAF (with respect to its *w*-estimation capability, neglecting its filtering component) with the classical RLS algorithm, we measured the mean squared errors of the *w*-estimate μ_*w*_, as shown in Figure 6 in a double-logarithmic plot over the number of time steps (shown is the average of the MSE-s of 1000 individual runs). In case of the PIAF algorithm, the variance estimate σ^2^_*w*_ is also shown (dashed line); it coincides well with the observed quadratic error. The process noise level was set to σ_*p*_ = 0.1, so the PIAF curves in Figure 6 correspond to Figures 4B,D.

The input to the classical RLS algorithm was given by the pairs ($\dot{q}$_*n*_, *x_n_* − *x*_*n*−1_) consisting of control values and the differences of consecutive observations. The difference of samples is according to Equations (1, 2) given by

(23) $${\dot{x}}_{n}=x_{n}-x_{n-1}=z_{n}-z_{n-1}+\epsilon_{s,n}-\epsilon_{s,n-1}=w\cdot \dot{q}+\epsilon_{\dot{x},n},$$

where the noise terms ϵ_ẋ,*n*_ are all identically distributed (but not independent) with variance 2σ^2^_*s*_ + σ^2^_*p*_. Since this difference signal is extremely noisy (in our setting the variance is more than 20 times larger than the amplitude of ẋ_*n*_, corresponding to a MSE SNR of approximately 1:1000), the initial estimates produced by RLS are very far away from the true value. The strength of the PIAF filter (seen as *w*-estimator) can be thus seen in the fact that it is not distracted by the noisiness of the samples and produces good *w*-estimates already early on. As Figure 6A shows, in the case of continuous control (as described in the previous subsection), RLS is nevertheless able to correct its wrong initial *w*-estimates. Within approximately 100 time steps its performance is henceforth comparable to that of the PIAF filter. The reason for that is that two consecutive errors ϵẋ,*n* and ϵẋ,*n*+1 are not independent: According to Equation (23) they both contain ϵ_*s,n*_; the first with a positive, the second with negative sign. If $\dot{q}$_*n*_ and $\dot{q}$_*n*+1_ are very similar, the noise term ϵ_*s,n*_ almost completely cancels out in the RLS' estimate for *w*.

In contrast, panel (B) shows that when the control signal $\dot{q}$_*n*_ is irregular, RLS cannot benefit from this “accidental” noise cancelation and its performance never catches up with PIAF.

### 4.3. Comparison with Kalman filtering

Finally, we compared the performance of PIAF (with respect to its *z*-estimation capability) with a classical Kalman filter that knew *w*. Note that PIAF must perform worse because it is not provided with the true value of *w*—but has to estimate it over time. The question is, if and how fast it can catch up.

**Figure 7:**
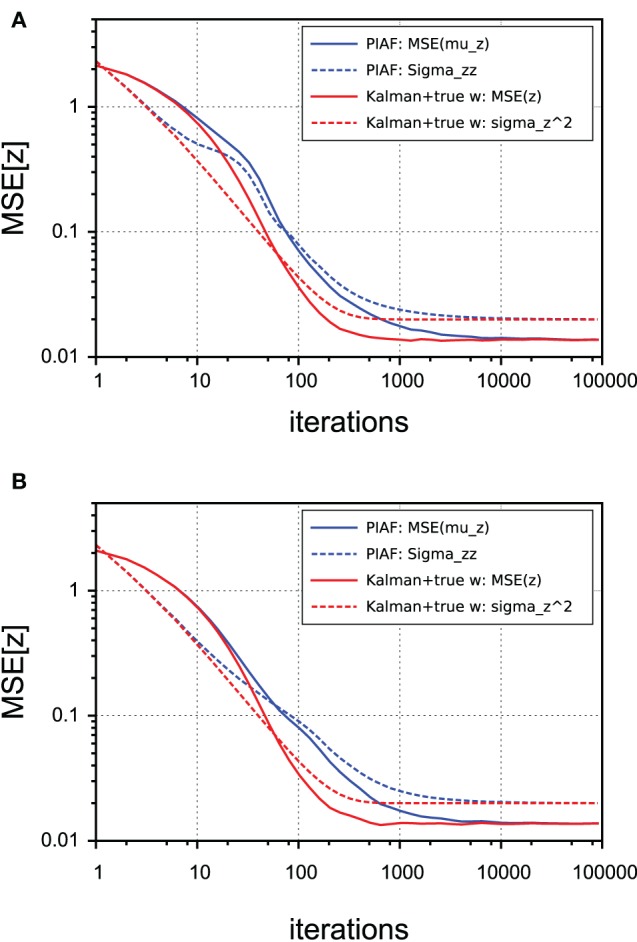
**Performance of *z*-estimation of PIAF (blue) and classical Kalman filter, but provided with knowledge of the true *w* (red)**. The accuracy is measured using the mean squared error. The variance estimates are shown using dashed lines. [σ_*s*_ = 2, σ_*p*_ = 0.01]. **(A)** Continuous control; **(B)** Random control.

In Figure 7 we show that irrespective of the type of control (continuous or random), the performance lags behind until the level of the process noise σ_*p*_ is reached, which corresponds to best possible performance. The lack of knowledge of the control weight *w* comes at a cost of requiring more samples to arrive at best possible accuracy. At an MSE level of 0.1, for example, the overhead to infer *w* amounted roughly to requiring twice the number of samples in our experiments.

As expected from the mathematical derivation, PIAF reaches the best possible performance, which is limited by the level of process noise σ^2^_*p*_. By comparing corresponding dotted lines (variance estimates of the algorithms) with the solid lines (actually observed mean squared error), it can be seen that in both cases the variance is initially under-estimated (by a factor of maximally 2) and finally a slightly overestimated (by a factor of ≈ 1.5). The difference between pairs of corresponding curves in Figure 7 is statistically significant in the intermediate range (from a few tens to a few thousands of iterations).

### 4.4. Comparing with *ad-hoc* combinations of Kalman and RLS

**Figure 8:**
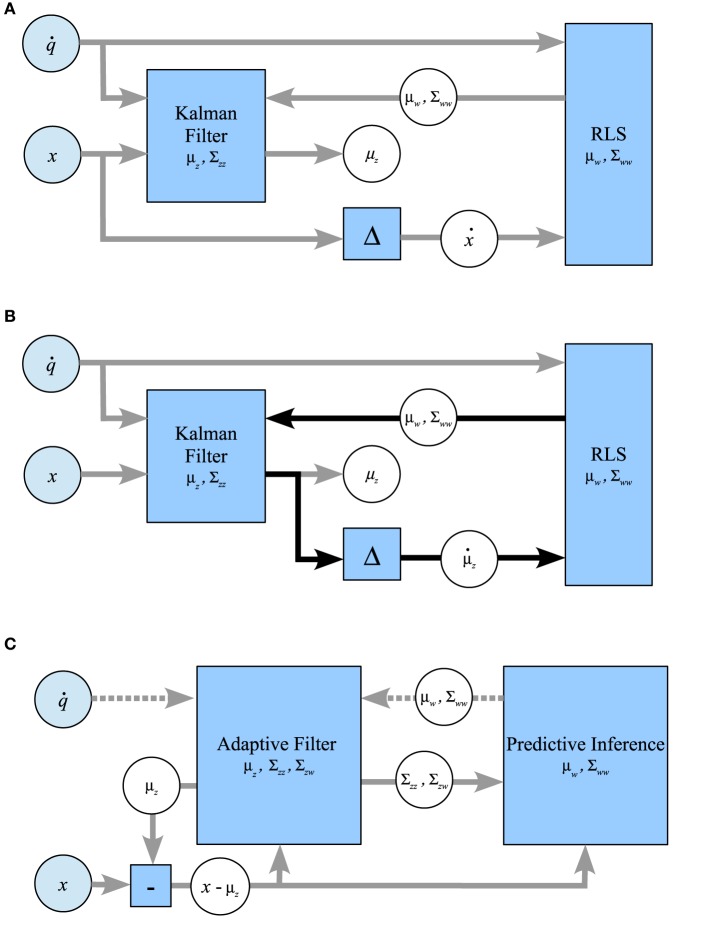
**Possible ways of combining the classical Kalman filtering algorithm and RLS in comparison with the combined system for prediction-based inference and adaptive filtering. (A)** RLS → Kalman: RLS obtains the difference of consecutive noisy measurements, Kalman is provided with *w*-estimates from RLS. **(B)** Kalman ↔ RLS: The Kalman filtered signal is used as input for RLS, which returns *w*-estimates. The feedback loop of this setup (indicated by black arrows) leads to self-delusional spiralling. **(C)** PIAF: Control signals and *w*-estimates are passed to the adaptive filter in the prediction step (dotted arrows). The deviation of the resulting prediction for the signal in the current time step μ_*z*_ from the new measurement x is used in the update step (solid arrows).

In this section, we consider some *ad-hoc* combinations of Kalman filtering and maximum likelihood estimation using RLS. Our aim is to show the drawbacks of these schemes in relation to Kalman filtering. Figure 8 sketches out the three considered systems schematically. Note that only PIAF exchanges posterior, conditional covariances (i.e., uncertainty) reciprocally between the state and forward model parameter estimations (cf. Figure 8C).

In the approach shown in Figure 8A, RLS is used to estimate *w* based on control signals $\dot{q}$_*n*_ and differences of consecutive unfiltered samples ẋ_*n*_ = *x_n_* − *x*_*n*−1_ (setting “RLS→Kalman”). In order to allow the Kalman filter to determine the optimal mixing coefficient, RLS must be extended to produce a variance estimate σ^2^_*w*_ in addition to its estimation of *w*. We use the expected standard error of the *w* estimate of RLS as calculated for example in Weisberg (2014) (see Supplementary Material for details). In this setup, RLS essentially assumes independent information sampling and cannot profit from the noise cancelation by the Kalman filter.

The second possibility (Figure 8B) attempts to benefit from Kalman filtering by using the difference of consecutive estimates μ_*z*_ produced by Kalman as the input to RLS (setting “Kalman↔RLS”). We will show that RLS will indeed initially learn slightly faster but will eventually end up in a delusional loop due to overconfidence in its learned forward model.

For comparison and illustration purposes, Figure 8C shows how PIAF can be split up into a subsystem for Adaptive Filtering (AF), during which the state estimate is adapted, and a subsystem for Predictive Inference (PI), during which the forward model is adjusted. This information loop resembles the one indicated by black arrows in Figure 8B. However, the information flow through the loop is augmented by variance estimates about the state estimates, the forward model parameter estimates, and their interdependencies: The AF component receives updates from the PI component about the forward model including the certainty about the forward model. Vice versa, the PI component receives updates from AF about the current state estimate, its certainty, and its interdependence with the forward model.

As a consequence, the control signal $\dot{q}$ is used only in the AF component directly. It is passed to the PI component indirectly via the prior variance and covariance estimates Σ_*zz,n*|*n*−1_ and Σ_*zw,n*|*n*−1_ and the residual between state signal and prior state estimate (*x_n_* − μ_*z,n*|*n*−1_). In this way, PIAF's adaptation of its forward model benefits from the current forward model knowledge and the interdependence of successive state signals, but it prevents overconfidence in its forward model-based state priors.

#### 4.4.1. Comparison with RLS→Kalman

**Figure 9:**
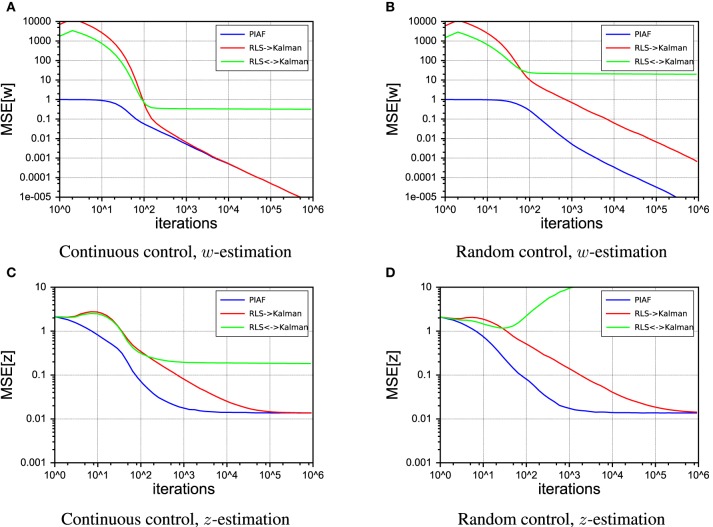
**Performance of the RLS → Kalman system (red, solid line), Kalman ↔ RLS (green, dashed lines) and PIAF (blue)**. [σ_*s*_ = 2, σ_*p*_ = 0.01]. **(A)** Continuous control, mean squared error of μ_*w*_, **(B)** Random control, mean squared error of μ_*w*_, **(C)** Continuous control, mean squared error of μ_*z*_, **(D)** Random control, mean squared error of μ_*z*_.

Compared with the straightforward *ad-hoc* system of Figures 8A, 9 shows that the PIAF system reduces the number of samples required to reach a certain target performance by a factor of 5–100 (horizontal offset between the curves PIAF and RLS→Kalman in Figure 9C). When applying random control commands, the RLS→Kalman system suffers further from an even slower convergence of the RLS-based forward model values ***w*** (cf. Figure 9B). As a consequence, Figure 9D shows that the *ad-hoc* combination has not yet reached the theoretically achievable target error performance even after 100k learning iterations (the theoretical optimum is bounded by σ_*p*_, as was also experimentally confirmed in Figure 5).

**Figure 10:**
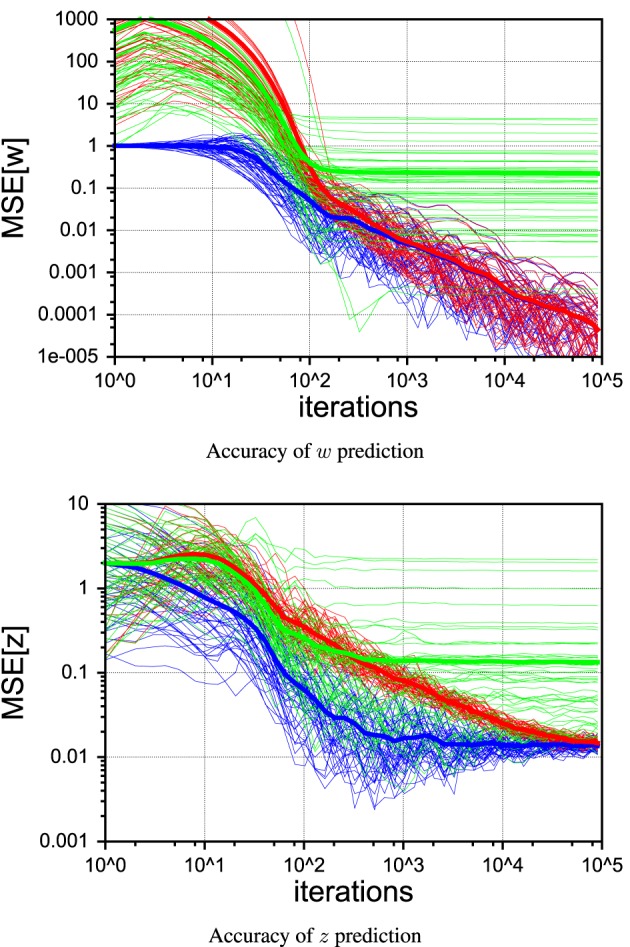
**Comparison of individual runs with continuous control of PIAF (blue) and RLS → Kalman (red) and Kalman ↔ RLS system (green)**. [σ_*s*_ = 2, σ_*p*_ = 0.01].

Figure 10 shows 50 individual runs of the PIAF system and the *ad-hoc* systems RLS→Kalman and Kalman↔RLS in the continuous control setup. All runs have identical settings, except for the seed value of the random generator that was used to produce the sensor and process noise samples. The average of each group of runs is shown by a thick line. While all runs of RLS→Kalman converge to the optimum, eventually, PIAF is significantly faster (compare with Figures 9A,C).

#### 4.4.2. The self-delusional loop: comparison with Kalman↔RLS

The *ad-hoc* system Kalman↔RLS of Figure 8B does not reach the theoretically achievable performance (determined by σ_*p*_). In the case of continuous control, it levels off at roughly 10 times higher mean squared error. In the case of random control signals, the estimates actually destabilize and estimated values become completely useless. This is due to the effect of the information feedback loop that leads to the self-delusional loop (cf. black arrows in Figure 8B). This insight was investigated in much more detail already elsewhere (Kneissler et al., 2012, 2014).

To illustrate the self-delusional loop further, the individual runs in Figure 10 show that the feedback loop of Kalman↔RLS can be initially advantageous for a certain fraction of runs, compared to RLS→Kalman and even reach the PIAF performance in a few cases. Nevertheless, sooner or later all of the Kalman↔RLS runs end up in stagnation, where the *w* estimates do not improve further. It is characteristic for the self-delusional loop that the stagnation occurs at an arbitrary point in time and the resulting end performances are widely spread.

## 5. Discussion

Any neural system that learns by predictive encoding principles inevitably faces the problem of learning to predict the effects of its own actions on itself and on its environment. Meanwhile, such a system will attempt to utilize current predictive knowledge to filter incoming sensory information—learning from the resulting residuals. In this paper, we have shown that a system can learn its forward model more than 10 times faster when using the filtered residual. However, we have also shown that a scheme composed of independent learning and prediction components with decoupled confidence estimation tends to become overly self-confident. When trapped in such a “delusional loop,” the system essentially overly trusts its internal forward model, disregarding residual information as noise and consequently prematurely preventing further learning.

To achieve the learning speed-up and to avoid the self-delusional loop, we have derived a Bayes-optimal solution to optimally combine the forward model knowledge with the incoming sensory feedback. The resulting Predictive Inference and Adaptive Filtering (PIAF) scheme learns the forward model and filters sensory information optimally, iteratively, and concurrently on the fly. PIAF was shown to be closely related to the recursive least squares (RLS) online linear regression technique as well as to Kalman filtering—combining both techniques in a Bayes-optimal manner by considering the covariances between the forward model parameters and the state estimates. In contrast to joint Kalman filtering approaches, which add the forward model parameters to the state estimate, PIAF separates the two components explicitly. Technically, PIAF rests on separating the exact posterior distribution over states and model parameters into these parameter groups. This statistical separation requires the exchange of sufficient statistics (in our Gaussian case, expectations, and covariances) between the Bayesian updates to ensure that uncertainty about the parameters informs state estimation and vice versa. The alternative would be to assume a joint posterior over both states and parameters and use a joint or global Kalman filter. However, this comes at a price of obfuscating the interaction between these two parameters.

Another generalization would be to repeat our experiments with a higher dimensional control space, where control commands $\dot{q}$_*n*_ are vectors. More importantly, at the moment the derivation is limited to linear models. In the non-linear case, Extended Kalman Filtering (EKF) methods or Unscented Kalman Filtering (UKF) techniques with augmented states are applicable. In our previous work, we have investigated locally linear mappings to approximate the underlying non-linear forward velocity kinematics model of a simulated robot arm (Kneissler et al., 2012, 2014), preventing self-delusional loops by means of thresholds. A general (Bayes optimal) solution for learning such locally linear mappings and possibly gain-field mappings, as identified in the brain in various cortical areas (Denève and Pouget, 2004; Chang et al., 2009), seems highly desirable. The main challenge in this respect is the estimation of dependencies between the forward model and the internal state estimates, when combining partially overlapping, locally linear forward model approximations and when traversing the local forward models. Our work shows that it is essential to prevent an overestimation of the forward model confidence—since overconfidence can lead to delusion. However, our work also shows that filtering the sensory signal and learning from the filtered signal is clearly worthwhile, because it has the potential to speed up learning by an order of magnitude and to provide more efficient inference.

### Conflict of interest statement

The authors declare that the research was conducted in the absence of any commercial or financial relationships that could be construed as a potential conflict of interest.

## Supplementary material

Please refer to the Supplementary Material for the full mathematical derivations and the relations to Kalman filtering and RLS.
