# Supplementary material for: Simultaneous learning and filtering without delusions: a Bayes-optimal combination of Predictive Inference and Adaptive Filtering
Source: Front Comput Neurosci. 2015 Apr 30;9:47. doi: 10.3389/fncom.2015.00047 (PMC4415408; doi:10.3389/fncom.2015.00047)
Supplement: Supplementary file 1 [file Presentation1.PDF]

Supplementary Material: Avoiding Self-Delusions: A Bayes-optimal Combination of Predictive Inference and Adaptive Filtering

Jan Kneissler [1,*], Jan Drugowitch [2] and Martin V. Butz [1]

[1] *Cognitive Modeling, Faculty of Science, Department of Computer Science, University of Tübingen, Tübingen, Germany*

[2] *Départment des Neurosciences Fondamentales, Université de Genève, Geneva, Switzerland*

Correspondence*:
Jan Kneissler
Cognitive Modeling, Faculty of Science, Department of Computer Science, University of Tübingen, Tübingen, Germany , jan.kneissler@uni-tuebingen.de

1 DERIVING THE MODEL UPDATE EQUATIONS

We assume the generative model given by:

$$z_0 \sim \mathcal{N}\left(\mu_{z,0|0}, \Sigma_{zz,0|0}\right), \tag{1}$$

$$\boldsymbol{w} \sim \mathcal{N}\left(\boldsymbol{\mu}_{w,0|0}, \boldsymbol{\Sigma}_{ww,0|0}\right), \tag{2}$$

$$z_n | z_{n-1}, \dot{\boldsymbol{q}}_n, \boldsymbol{w} \sim \mathcal{N}\left(z_{n-1} + \dot{\boldsymbol{q}}_n^T \boldsymbol{w}, \sigma_p^2\right), \tag{3}$$

$$x_n | z_n \sim \mathcal{N}\left(z_n, \sigma_s^2\right), \tag{4}$$

The joint posterior $p(z, \boldsymbol{w}| \dots)$ has the following parameters:

$$\left(\begin{pmatrix} z \\ \boldsymbol{w} \end{pmatrix} \Big| \dots \right) = \mathcal{N}\left(\begin{pmatrix} \mu_z \\ \boldsymbol{\mu}_w \end{pmatrix}, \begin{pmatrix} \Sigma_{zz} & \boldsymbol{\Sigma}_{zw} \\ \boldsymbol{\Sigma}_{zw}^T & \boldsymbol{\Sigma}_{ww} \end{pmatrix} \right), \tag{5}$$

with the following covariance matrix block structure and its inverse:

$$\boldsymbol{\Sigma} = \begin{pmatrix} \Sigma_{zz} & \boldsymbol{\Sigma}_{zw} \\ \boldsymbol{\Sigma}_{zw}^T & \boldsymbol{\Sigma}_{ww} \end{pmatrix}, \qquad \boldsymbol{\Lambda} \equiv \boldsymbol{\Sigma}^{-1} = \begin{pmatrix} \Lambda_{zz} & \boldsymbol{\Lambda}_{zw} \\ \boldsymbol{\Lambda}_{zw}^T & \boldsymbol{\Lambda}_{ww} \end{pmatrix}. \tag{6}$$

The blocks represent the variance Σ_{zz} of the estimate of z, the D_q-element covariance row vector $\boldsymbol{\Sigma}_{zw}$ between z and $\boldsymbol{w}$, and the $D_q \times D_q$ covariance matrix $\boldsymbol{\Sigma}_{ww}$ of $\boldsymbol{w}$. With this, the marginal estimates for z and $\boldsymbol{w}$ are given by $p(z|\mu_z, \Sigma_{zz}) = \mathcal{N}(z|\mu_z, \Sigma_{zz})$ and $p(\boldsymbol{w}|\boldsymbol{\mu}_w, \boldsymbol{\Sigma}_{ww}) = \mathcal{N}(\boldsymbol{w}|\boldsymbol{\mu}_w, \boldsymbol{\Sigma}_{ww})$. As it will be of use later, the relevant terms of the joint density of z and $\boldsymbol{w}$ are given by

$$p(z, \boldsymbol{w}| \dots) \underset{z,\boldsymbol{w}}{\propto} e^{-\frac{1}{2}\Lambda_{zz} z^2 - \frac{1}{2}\boldsymbol{w}^T \boldsymbol{\Lambda}_{ww} \boldsymbol{w} - \boldsymbol{\Lambda}_{zw} \boldsymbol{w} z + (\Lambda_{zz}\mu_z + \boldsymbol{\Lambda}_{zw}\boldsymbol{\mu}_w) z + (\mu_z \boldsymbol{\Lambda}_{zw} + \boldsymbol{\mu}_w^T \boldsymbol{\Lambda}_{ww}) \boldsymbol{w}}. \tag{7}$$

Inference is split into the prediction step and the update step, causing parameter changes as described in the main text.

1.1 PREDICTION STEP

The prediction step requires handling the transition from z_{n-1} to z_n as well as including knowledge about the applied motor command $\dot{\boldsymbol{q}}_n$. Before the prediction step, the joint $p(z_{n-1}, \boldsymbol{w}|x_{1:n-1}, \dot{\boldsymbol{q}}_{1:n-1})$ has parameters $\mu_{z,n-1|n-1}$, $\boldsymbol{\mu}_{w,n-1|n-1}$, $\Sigma_{zz,n-1|n-1}$, $\boldsymbol{\Sigma}_{zw,n-1|n-1}$, and $\boldsymbol{\Sigma}_{ww,n-1|n-1}$. The prediction step is given by the marginalization

$$p(z_n, \boldsymbol{w}|x_{1:n-1}, \dot{\boldsymbol{q}}_{1:n-1}) = \int p(z_n|z_{n-1}, \dot{\boldsymbol{q}}_n, \boldsymbol{w}, \sigma_p^2) p(z_{n-1}, \boldsymbol{w}|x_{1:n-1}, \dot{\boldsymbol{q}}_{1:n-1}) \mathrm{d}z_{n-1}. \quad (8)$$

Evaluating this integral directly is tedious due to the need to invert block-structured matrices. Instead, we will use the knowledge that the posterior is Gaussian, such that it is sufficient to evaluate its first and second-order moments. These moments are found by using the following transition:

$$z_n = z_{n-1} + \dot{\boldsymbol{q}}_n^T \boldsymbol{w} + \epsilon_{p,n}, \quad (9)$$

resulting in the posterior mean z_n,

$$\langle z_n \rangle = \langle z_{n-1} \rangle + \dot{\boldsymbol{q}}_n^T \langle \boldsymbol{w} \rangle. \quad (10)$$

Knowing $\dot{\boldsymbol{q}}_n$ does not provide any information about $\boldsymbol{w}$, such that its mean remains unchanged. The covariances are found by again using Eq. (9), and result in

$$\begin{aligned} \mathrm{var}(z_n) &= \langle \left(z_{n-1} + \dot{\boldsymbol{q}}_n^T \boldsymbol{w} + \epsilon_p - \langle z_{n-1} \rangle - \dot{\boldsymbol{q}}_n^T \langle \boldsymbol{w} \rangle \right)^2 \rangle \quad (11) \\ &= \mathrm{var}(z_{n-1}) + \dot{\boldsymbol{q}}_n^T \,\mathrm{cov}(\boldsymbol{w}, \boldsymbol{w}) \dot{\boldsymbol{q}}_n + \mathrm{var}(\epsilon_p) + 2\,\mathrm{cov}(z_{n-1}, \boldsymbol{w}^T) \dot{\boldsymbol{q}}_n \\ \mathrm{cov}(z_n, \boldsymbol{w}^T) &= \langle \left(z_{n-1} + \dot{\boldsymbol{q}}_n^T \boldsymbol{w} + \epsilon_p - \langle z_{n-1} \rangle - \dot{\boldsymbol{q}}_n^T \langle \boldsymbol{w} \rangle \right) \left(\boldsymbol{w}^T - \langle \boldsymbol{w} \rangle^T \right) \rangle \\ &= \mathrm{cov}(z_{n-1}, \boldsymbol{w}^T) + \dot{\boldsymbol{q}}_n^T \,\mathrm{cov}(\boldsymbol{w}, \boldsymbol{w}). \quad (12) \end{aligned}$$

The covariance of $\boldsymbol{w}$ remains unchanged. The parameter changes in the prediction step of the adaptive filter result from substituting the means, variances, and covariances in the above with the parameters of $p(z_n, w|x_{1:n-1}, \dot{\boldsymbol{q}}_{1:n-1})$.

1.2 UPDATE STEP

The update steps includes knowledge of x_n in the current estimate $p(z_n, \boldsymbol{w}|x_{1:n-1}, \dot{\boldsymbol{q}}_{1:n})$, by applying Bayes' rule,

$$p(z_n, \boldsymbol{w}|x_{1:n}, \dot{\boldsymbol{q}}_{1:n}) \propto p(x_n|z_n) p(z_n, \boldsymbol{w}|x_{1:n-1}, \dot{\boldsymbol{q}}_{1:n}). \quad (13)$$

To find the parameter update equations, denote $\boldsymbol{m} = \boldsymbol{\mu}_{n|n-1}$ and $\boldsymbol{P} = \boldsymbol{\Lambda}_{n|n-1}$, and expand the above to find

$$\begin{aligned} &p(z_n, \boldsymbol{w}|x_{1:n}, \dot{\boldsymbol{q}}_{1:n}) \quad (14) \\ &\propto e^{-\frac{1}{2}\left(P_{zz}+\frac{1}{\sigma_s^2}\right) z_n^2 - \frac{1}{2}\boldsymbol{w}^T \boldsymbol{P}_{ww} \boldsymbol{w} - \boldsymbol{P}_{zw} \boldsymbol{w} z_n + \left(P_{zz} m_z + \boldsymbol{P}_{zw} \boldsymbol{m}_w + \frac{x_n}{\sigma_s^2}\right) z_n + (m_z \boldsymbol{P}_{zw} + \boldsymbol{m}_w^T \boldsymbol{P}_{ww}) \boldsymbol{w}}. \end{aligned}$$

Comparing the above with Eq. (7) allows us to read off the updated precision matrix

$$\boldsymbol{\Lambda}_{n|n} = \begin{pmatrix} P_{zz} + \frac{1}{\sigma_s^2} & \boldsymbol{P}_{zw} \\ \boldsymbol{P}_{zw}^T & \boldsymbol{P}_{ww}^T \end{pmatrix} \quad (15)$$

Furthermore, the posterior means need to satisfy the following equations,

$$P_{zz}m_z + \boldsymbol{P}_{zw}\boldsymbol{m}_w + \frac{x_n}{\sigma_s^2} = \Lambda_{zz,n|n}\mu_{z,n|n} + \boldsymbol{\Lambda}_{zw,n|n}\boldsymbol{\mu}_{w,n|n}, \tag{16}$$

$$m_z\boldsymbol{P}_{zw} + \boldsymbol{m}_w^T\boldsymbol{P}_{ww} = \mu_{z,n|n}\boldsymbol{\Lambda}_{zw,n|n} + \boldsymbol{\mu}_{w,n|n}^T\boldsymbol{\Lambda}_{ww,n|n}. \tag{17}$$

We find the posterior covariance matrix by using the matrix inversion lemma to invert the precision matrix, resulting in

$$\boldsymbol{\Sigma}_{n|n} = \begin{pmatrix} \frac{\sigma_s^2}{\sigma_s^2 P_{zz}+1-\sigma_s^2\boldsymbol{P}_{zw}\boldsymbol{P}_{ww}^{-1}\boldsymbol{P}_{zw}^T} & -\frac{\sigma_s^2\boldsymbol{P}_{zw}\boldsymbol{P}_{ww}^{-1}}{\sigma_s^2 P_{zz}+1-\sigma_s^2\boldsymbol{P}_{zw}\boldsymbol{P}_{ww}^{-1}\boldsymbol{P}_{zw}^T} \\ -\frac{\sigma_s^2\boldsymbol{P}_{ww}^{-1}\boldsymbol{P}_{zw}^T}{\sigma_s^2 P_{zz}+1-\sigma_s^2\boldsymbol{P}_{zw}\boldsymbol{P}_{ww}^{-1}\boldsymbol{P}_{zw}^T} & \boldsymbol{P}_{ww}^{-1} + \frac{\sigma_s^2\boldsymbol{P}_{ww}^{-1}\boldsymbol{P}_{zw}^T\boldsymbol{P}_{zw}\boldsymbol{P}_{ww}^{-1}}{\sigma_s^2 P_{zz}+1-\sigma_s^2\boldsymbol{P}_{zw}\boldsymbol{P}_{ww}^{-1}\boldsymbol{P}_{zw}^T} \end{pmatrix}. \tag{18}$$

Denoting $\boldsymbol{S} = \Sigma_{n|n-1}$ and utilizing once more the matrix inversion lemma the following relationships can be derived

$$S_{zz}^{-1} = P_{zz} - \boldsymbol{P}_{zw}\boldsymbol{P}_{ww}^{-1}\boldsymbol{P}_{zw}^T, \tag{19}$$

$$-S_{zz}^{-1}\boldsymbol{S}_{zw} = \boldsymbol{P}_{zw}\boldsymbol{P}_{ww}^{-1}, \tag{20}$$

$$\boldsymbol{S}_{ww} - \boldsymbol{S}_{zw}^T S_{zz}^{-1}\boldsymbol{S}_{zw} = \boldsymbol{P}_{ww}^{-1}, \tag{21}$$

which we can turn into an expression of the previous covariance matrix, resulting in

$$\boldsymbol{\Sigma}_{n|n} = \begin{pmatrix} \frac{\sigma_s^2}{\sigma_s^2+S_{zz}}S_{zz} & \frac{\sigma_s^2}{\sigma_s^2+S_{zz}}\boldsymbol{S}_{zw} \\ \frac{\sigma_s^2}{\sigma_s^2+S_{zz}}\boldsymbol{S}_{zw}^T & \boldsymbol{S}_{ww} - \frac{\boldsymbol{S}_{zw}^T\boldsymbol{S}_{zw}}{\sigma_s^2+S_{zz}} \end{pmatrix}. \tag{22}$$

In order to find the posterior means, we need to use the further relationships

$$\boldsymbol{P}_{zw} = -P_{zz}\boldsymbol{S}_{zw}\boldsymbol{S}_{ww}^{-1}, \tag{23}$$

$$P_{zz} = (S_{zz} - \boldsymbol{S}_{zw}\boldsymbol{S}_{ww}^{-1}\boldsymbol{S}_{zw}^T)^{-1}. \tag{24}$$

Based on these, we can solve the coupled equations for $\mu_{z,n|n}$ and $\boldsymbol{\mu}_{w,n|n}$ to find

$$\mu_{z,n|n} = m_z + \frac{S_{zz}}{\sigma_s^2 + S_{zz}}(x_n - m_z), \tag{25}$$

$$\boldsymbol{\mu}_{w,n|n} = \boldsymbol{m}_w + \frac{\boldsymbol{S}_{zw}^T}{\sigma_s^2 + S_{zz}}(x_n - m_z). \tag{26}$$

Combining all of the above results in the parameter updates described in the adaptive filtering updates and the predictive inference updates of the main text.

2 KALMAN FILTER AND RLS UPDATE EQUATIONS

2.1 KALMAN FILTER

In contrast to our model (Eqs. (1)-(4)), the Kalman filter assumes full knowledge of $\boldsymbol{w}$. In this case, the prediction step is found as before, by computing the first and second-order moments of z_n, based on

Eq. (9). This results in the parameter updates given in the main text. The update step for z_n follows from Bayes' rule,

$$\begin{aligned} & p(z_n|x_{1:n}, \dot{\boldsymbol{q}}_{1:n}, \boldsymbol{w}) \\ & \quad \propto p(x_n|z_n) p(z_n|x_{1:n-1}, \dot{\boldsymbol{q}}_{1:n}, \boldsymbol{w}) \\ & \quad \propto \mathcal{N}\left(z_n|\mu_{z,n|n-1} + \frac{\Sigma_{zz,n|n-1}}{\sigma_s^2 + \Sigma_{zz,n|n-1}}(x_n - \mu_{z,n|n-1}), \frac{\sigma_s^2 \Sigma_{zz,n|n-1}}{\sigma_s^2 + \Sigma_{zz,n|n-1}} \right), \end{aligned} \tag{27}$$

resulting in the parameter updates given in the main text.

2.2 RECURSIVE LEAST-SQUARES

As described in the main text, we apply Recursive Least Squares (RLS) to $\dot{x}_1, \dot{x}_2, \ldots$, while ignoring that sequential $\dot{x}_n$'s are correlated. The $\dot{x}_n$'s themselves relate to $\boldsymbol{w}$ by $\dot{x}_n = x_n - x_{n-1} = \dot{z}_n + 2\epsilon_{s,n}$ (instead of $\dot{x}_n = \dot{z}_n + \epsilon_{s,n} - \epsilon_{s,n-1}$ for the full model) and $\dot{z}_n = z_n - z_{n-1} = \dot{\boldsymbol{q}}_n^T \boldsymbol{w} + \epsilon_{p,n}$.

In the prediction step we observe $\dot{\boldsymbol{q}}_n$ without knowing $\dot{x}_n$. As $\dot{\boldsymbol{q}}_n$ does not provide any information about $\boldsymbol{w}$, its associated belief parameters remain unchanged. However, it will couple $\dot{z}_n$ to $\boldsymbol{w}$, and will cause the belief over $\boldsymbol{w}$ to predict what $\dot{z}_n$ ought to be under this belief for the applied motor command. Assuming $\boldsymbol{\mu}_w$ and $\boldsymbol{\Sigma}_{ww}$ to denote the parameters of the $\boldsymbol{w}$ estimate before the prediction step, we find the joint $\dot{z}_n$ and $\boldsymbol{w}$ by Bayes' rule,

$$\begin{aligned} p(\dot{z}_n, \boldsymbol{w}|\dot{\boldsymbol{q}}_n, \ldots) & \propto p(\dot{z}_n|\dot{\boldsymbol{q}}_n, \boldsymbol{w}) p(\boldsymbol{w}|\boldsymbol{\mu}_w, \boldsymbol{\Sigma}_{ww}) \\ & = \mathcal{N}(\dot{z}_n|\dot{\boldsymbol{q}}_n^T \boldsymbol{w}, \sigma_p^2) \mathcal{N}(w|\boldsymbol{\mu}_w, \boldsymbol{\Sigma}_{ww}) \\ & \propto e^{-\frac{1}{2\sigma_p^2}\dot{z}_n^2 - \frac{1}{2}\boldsymbol{w}^T\left(\boldsymbol{\Sigma}_{ww}^{-1} + \frac{1}{\sigma_p^2}\dot{\boldsymbol{q}}_n\dot{\boldsymbol{q}}_n^T\right)\boldsymbol{w} + \frac{\dot{\boldsymbol{q}}_n^T}{\sigma_p^2}\boldsymbol{w}\dot{z}_n + \boldsymbol{\mu}_w^T \boldsymbol{\Sigma}_{ww}^{-1}} \\ & \propto \mathcal{N}((\dot{z}_n, \boldsymbol{w}^T)^T | (\dot{\mu}_z, \boldsymbol{\mu}_w^T)^T, \Sigma), \end{aligned} \tag{28}$$

where we have used $\dot{\mu}_z$ to indicate that this mean represents the mean change in z rather than the mean of z. We find the precision matrix of the posterior by relating the above to Eq. (7), and the associated covariance matrix by applying the matrix inversion lemma, resulting in

$$\boldsymbol{\Lambda} = \begin{pmatrix} \frac{1}{\sigma_p^2} & -\frac{1}{\sigma_p^2}\dot{\boldsymbol{q}}_n^T \\ -\frac{1}{\sigma_p^2}\dot{\boldsymbol{q}}_n & \boldsymbol{\Sigma}_{ww}^{-1} + \frac{1}{\sigma_p^2}\dot{\boldsymbol{q}}_n\dot{\boldsymbol{q}}^T \end{pmatrix}, \qquad \boldsymbol{\Sigma} = \begin{pmatrix} \sigma_p^2 + \dot{\boldsymbol{q}}_n^T \boldsymbol{\Sigma}_{ww} \dot{\boldsymbol{q}}_n & \dot{\boldsymbol{q}}_n^T \boldsymbol{\Sigma}_{ww} \\ \boldsymbol{\Sigma}_{ww}\dot{\boldsymbol{q}}_n & \boldsymbol{\Sigma}_{ww} \end{pmatrix}. \tag{29}$$

The means are found in the same way, resulting in

$$\dot{\mu}_z = \dot{\boldsymbol{q}}_n^T \boldsymbol{\mu}_w, \qquad \boldsymbol{\mu}_w = \boldsymbol{\mu}_w. \tag{30}$$

We relate these equations to the prediction step of the full model by setting $\mu_{z,n|n-1} = \mu_{z,n-1|n-1} + \dot{\mu}_z$, $\boldsymbol{\Sigma}_{ww,n-1|n-1} = \boldsymbol{\Sigma}_{ww}$ and $\boldsymbol{\mu}_{w,n-1|n-1} = \boldsymbol{\mu}_0$, and update $\Sigma_{zz,n-1|n-1}$ to $\Sigma_{zz,n|n-1}$ by adding the covariance associated with $\dot{z}$. This substitution results in the same prediction step as for the full model, except for the update for Σ_{zz} and $\boldsymbol{\Sigma}_{zw}$, both of which are provided in the main text.

In the update step, we include knowledge of $\dot{x}_n$ in the posterior over $\dot{z}_n$ and $\boldsymbol{w}$. This is done by Bayes' rule, exactly as for the update step of our full model. As a consequence, the update equations of the individual parameters are the same as those for the full model, only that σ_s^2 is now replaced by $2\sigma_s^2$.

To show that this algorithm is indeed the RLS algorithm, we can combine the update and the prediction step, and assume that $\boldsymbol{\Sigma}_{ww,n-1|n-1} = \boldsymbol{\Sigma}_{ww}$ and $\boldsymbol{\mu}_{w,n-1|n-1} = \boldsymbol{\mu}_w$. Then, we find that

$$\boldsymbol{\alpha}_n = \frac{\boldsymbol{\Sigma}_{ww}\dot{\boldsymbol{q}}_n}{2\sigma_s^2 + \sigma_p^2 + \dot{\boldsymbol{q}}_n^T\boldsymbol{\Sigma}_{ww}\dot{\boldsymbol{q}}_n}, \tag{31}$$

$$\boldsymbol{\mu}_{w,n|n} = \boldsymbol{\mu}_w + \boldsymbol{\alpha}_n(\dot{x}_n - \dot{\boldsymbol{q}}_n^T\boldsymbol{\mu}_w), \tag{32}$$

$$\boldsymbol{\Sigma}_{ww,n|n} = \boldsymbol{\Sigma}_{ww} - \boldsymbol{\alpha}_n\dot{\boldsymbol{q}}_n^T\boldsymbol{\Sigma}_{ww}, \tag{33}$$

which corresponds to a single RLS update with observation variance $2\sigma_s^2 + \sigma_p^2$.

3 RLS WITH VARIANCE ESTIMATE

The RLS→Kalman system, whose performance is compared with PIAF in the evaluation section of the main text, uses a classical one-dimensional RLS system given by the update equations

$$\epsilon_n = \dot{x}_n - \dot{q}_n^T w_{n-1}, \tag{34}$$

$$g_n = \frac{P_{n-1}\dot{q}_n}{\lambda + \dot{q}_n^T P_{n-1}\dot{q}_n}, \tag{35}$$

$$w_n = w_{n-1} + \epsilon_n g_n, \tag{36}$$

$$P_n = \lambda^{-1}P_{n-1} - g_n\dot{q}_n^T\lambda^{-1}P_{n-1}. \tag{37}$$

The resulting w-estimates $\hat{w}$ optimize the cost function $\sum_{i=0}^{n}\lambda^{n-i}\left(\dot{x}_i - \dot{q}_i\hat{w}\right)^2$.

The corresponding variance estimate is given by $\widehat{Var}(\hat{w}) = \frac{\hat{\sigma}^2}{\mathrm{SXX}}$. In order to obtain an online algorithm, we approximated the variance $\hat{\sigma}^2$ by $\frac{\sum_{i=0}^{n}\lambda^{n-i}\epsilon_i^2}{\sum_{i=0}^{n}\lambda^{n-i}}$ and computed the denominator according to

$$\mathrm{SXX} = \sum_{i=0}^{n}\lambda^{n-i}\left(\dot{q}_i - \bar{\dot{q}}\right)^2 = \sum_{i=0}^{n}\lambda^{n-i}\dot{q}_i^2 - \left(\sum_{i=0}^{n}\lambda^{n-i}\dot{q}_i\right)^2 / \left(\sum_{i=0}^{n}\lambda^{n-i}\right), \tag{38}$$

which leads to the following update equations:

$$\mathrm{E}_n = \lambda E_{n-1} + \epsilon_n, \tag{39}$$

$$\mathrm{Q}_n = \lambda Q_{n-1} + \dot{q}_n, \tag{40}$$

$$\mathrm{S}_n = \lambda S_{n-1} + \dot{q}_n^2, \tag{41}$$

$$\mathrm{C}_n = \lambda C_{n-1} + 1. \tag{42}$$

The estimate for the w variance in time step n is then given by

$$\hat{\sigma}_{w,n}^2 = \frac{E_n}{S_n C_n - Q_n^2}. \tag{43}$$
